# Memorandum by the Council on the Petition to the Prime Minister

**Published:** 1917-06-02

**Authors:** 


**To the Members of the College of Nursing, Ltd.**

### MEMORANDUM BY THE COUNCIL ON THE PETITION TO THE PRIME MINISTER

PROMOTED BY THE

### SOCIETY for the STATE REGISTRATION of TRAINED NURSES.

It has been pointed out to the Council that some pronouncement is desirable as to the official attitude of the College towards the above Petition, which urges that "any Bill for the State Registration of "Nurses, brought in by the Government, or other-"wise, shall provide for the direct representation of "the Organised Societies of Nurses on the Pro-"visional Governing Body" . . . "as provided "in the Bill drafted by the Central Committee for "the State Registration of Nurses."

A Considerable Danger.

Whilst it is in the highest degree improbable that "the Government" can, at the moment, bring in a Bill for State Registration, there is considerable danger that a claim may be made that any signatures given to the Petition, though in reality given only in favour of the one point of "direct representation," really imply a general support of the Central Committee's Bill as against the Bill promoted by the College.

How the Two Bills Differ.

It becomes, therefore, necessary to consider the principal differences between the two measures.

Both Bills set out to promote State Registration of Trained Nurses by Act of Parliament; a three years' term of grace for practising Nurses after the Bill becomes law, during which time they can register without further examination; a uniform curriculum (or course of training) at recognised Hospitals and Infirmaries; a central examination for all Nurses at the expiration of the three years' term of grace.

Both Bills constitute what the Petition describes as a Provisional Governing Body (a Provisional Council), which is to frame rules for the establishment of the ultimate or Permanent Council that is to regulate the Profession. But there is this difference, that, whereas the College Bill lays it down that on the Permanent Council registered nurses are to elect not fewer than two-thirds of the members, under the Bill of the Central Committee they elect only sixteen out of thirty-three members, and eight of these must be "past or present Matrons of Nurse-Training Schools."

How the College Bill was Developed.

Framed at first in a tentative manner the College Bill has been submitted from time to time to those best able to advise as to the intricacies of Parliamentary procedure. It has been moulded by their counsel, and even now has not reached its final form. What, however, these authorities have been from the first unanimous in urging is that, if in the present condition of public business the Bill is to have any chance of passing through Parliament, it must be as short as possible, must stick to essential matters, and must avoid details. This is what the College Bill seeks to do, and the Central Committee's Bill certainly fails to do.

It is true that in the earliest draft of the College Bill the Provisional Council was to be appointed as to one-third by the Privy Council, Government Departments, and the Medical Profession; as to one-third by the Council of the College of Nursing; and as to one-third by the Central Committee for the State Registration of Nurses. In the next draft the Provisional Council was to be appointed as to one-third by the British Medical Association; as to one-third by the Council of the College; and as to one-third by the Central Committee. But the more carefully these conditions were scrutinised the more evident it became that they would fail to facilitate the passing of the Bill. Insistent demands for direct representation were speedily put forward on behalf of the Medico-Psychological Association, on behalf of various bodies claiming to represent the interests of Poor-Law Authorities and Poor-Law nurses, on behalf of the authorities of Nurse-Training Schools, and on behalf of Scotland and Ireland.

An Agreed Bill Essential.

The Council thus became convinced that this clause, as it stood, would lead to endless trouble in Parliament as to what bodies and interests should be included and what excluded from direct representation, and to what proportional representation they were entitled. On the other hand they believed, and still believe, all these matters to be quite capable of satisfactory adjustment by conference amongst the parties concerned and the "give and take" which is essential for the production of an Agreed Bill dealing with questions which have aroused, and still arouse, such acute differences of opinion in the Nursing profession and outside.

M.P.'s Difficulties.

Moreover, they could not disguise from themselves that the average Member of Parliament is quite ignorant of the existence of many of the "Societies of Organised Nurses," some of which are of recent growth, and even of the College of Nursing and of the Central Committee itself, and that for these and other reasons he would be more likely to vote for a Registration Bill which set out the actual names of the Provisional Council than if

(*Continued on p.* 176.)

### MEMORANDUM BY THE COUNCIL OF THE COLLEGE OF NURSING, LTD. (*Continued from p. 174.*)

he were asked to entrust the nomination of it to bodies of which his constituents and himself knew little or nothing.

### No Wish to Ignore Central Committee.

And here the Council wishes to emphasise the fact that the College in framing its Registration Bill, and naming the Provisional Council, has no wish to ignore the Central Committee and the Organised Nurses' Societies. It still hopes to give them adequate representation on the Provisional Council, but it is convinced that it is more politic to attempt to conciliate all conflicting interests which claim representation thereon, by negotiation and compromise before going to Parliament, than to confront the House with a vexed problem which Members generally will have neither the time to debate nor the knowledge to solve.

Hence the preference by the College for a named and agreed list of the Provisional Council rather than the alternative proposal of the Central Committee, which inserts in the Bill just a list of nominating Bodies, obviously contentious and incomplete. As Mr. Stanley has said: "Both parties are out for State Registration. It is merely a question of how to get it with a minimum of friction and delay."

### No Substantial Progress before the College Came.

The Bill of the Central Committee on its present lines has been before Parliament now for many years, and no substantial progress has been made. Is it not time that other methods should be tried? Such, at least, is the opinion of the Council, and, therefore, members of the College are advised Not to Sign the Petition, but to leave the Council of the College free to promote its Bill for the State Registration of Nurses in whatever form may seem most likely to conduce to its speedy acceptance by Parliament.

### The Advantages the College Offers over Other Schemes.

If the admitted object of all State Registrationists is to expedite the time when Nurses can individually take part in electing their own Council, attention may be drawn to the following points in which the College Bill appears to possess important advantage over that promoted by the Central Committee.

The Bill promoted by the College makes the Register of the College the first Legal Register, and the Provisional Council has nothing to do except to frame rules and regulations to put the Act in force —a task which, with the approval of the Privy Council, can be readily carried out in even less than the period of two years to which the life of the Provisional Council is limited. Then, at once, this first Council retires to give place to the Permanent Council, two-thirds of which, as above stated, will be elected by the Nurses on the Register themselves —the only form of *direct* representation which Nurses have a vital interest in securing as speedily as may be.

### Not a Nurse can be put on the Legal Register.

On the other hand, under the Bill promoted by the Central Committee, not a Nurse can be put on the Legal Register until the Provisional Council has formulated the Rules under which existing Nurses may register, and has had them sanctioned by the Privy Council. Moreover, such Nurses as register at once get no chance of proceeding to elect their representatives on the Permanent Council until enough of them have registered, and enough Training Schools have received recognition, for the Lord President of the Council to certify that the task of forming the Register and recognising the Schools "is sufficiently advanced to admit of the election."

If, under these conditions, Training Schools decline to apply for recognition, and existing Nurses do not think it worth while to register, or, as well may happen, defer their registration to the end of the three years of grace, waiting to see how things are going, we may find the Provisional Council in possession, and, incidentally, enjoying the advantages of possession, for a period to be reckoned by years rather than months, and for the whole of this time Nurses will be deprived of their privilege to elect even such a fraction of Nurse Representatives on the Permanent Council as the Bill gives them.

### Members not to Sign the Petition.

The Bill of the College has not reached its final revision. It is still capable of improvement, and will be improved; but from what has been said Members will judge that the Council strongly advises them Not to Sign the Petition. To sign it will be claimed as supporting the policy of the Central Committee against the College, and will certainly tend to hamper the Council in conducting the complicated negotiations still to be undertaken with other bodies before it is in a position to present such a Bill for the State Registration of Nurses to Parliament as, commanding general acceptance, may speedily find a place in the Statute-book.

M. S. Rundle, Secretary.

6 Vere Street, London, W. 1, May 24, 1917.

### TO MEMBERS OF THE COLLEGE OF NURSING, LTD.

### IMPORTANT.

You are advised by the Council of the College *not to sign* the Petition which has been sent to you by the Society for the State Registration of Trained Nurses. If under a misapprehension you have already done so, you should write immediately to withdraw your name.

*This intimation accompanied the above statement, which gives in detail the reasons for this word of caution.*

